# Supplementary material for: The Evolution and Characterization of the RNA Interference Pathways in Lophotrochozoa
Source: Genome Biol Evol. 2024 May 7;16(5):evae098. doi: 10.1093/gbe/evae098 (PMC11114477; doi:10.1093/gbe/evae098)
Supplement: evae098_Supplementary_Data [file evae098_supplementary_data.zip › Supplementary_materials.zip!/selected_miRNAs/NoGe_selected_miRNAs/known_miRNA_families_pdf_files/NMRB01000131.1_Notospermus_geniculatus_strain_Ushimado_scaffold131,_whole_genome_shotgun_sequence_5275.pdf]

Provisional ID	:	NMRB01000131.1_Notospermus_geniculatus_strain_Ushimado_scaffold131,_whole_genome_shotgun_sequence_5275
Score total	:	221374.3
Score for star read(s)	:	3.9
Score for read counts	:	221365.7
Score for mfe	:	1.7
Score for randfold	:	0
Score for cons. seed	:	3
Total read count	:	434211
Mature read count	:	433367
Loop read count	:	0
Star read count	:	844

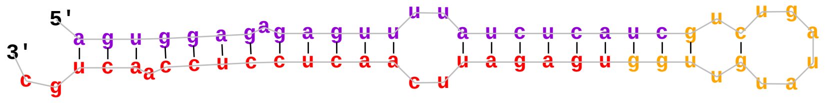

Genome coordinates: NMRB01000131.1 555868-555928

pre-miRNA sequence:
AGUGGAGAGAGUUUUAUCUCAUCAGUGGAGAGAGUUUUAUCUCAUCGUCUGAUAUGUUGGUGAGAUUCAACUCCUCCAACUGC

Mature sequence: UGAGAUUCAACUCCUCCAACUGC

Aligned mature sequence of the *N. geniculatus* pre-miRNA with the top 5 hits from the BLAST search against the pre-miRNA mirGeneDB set:

```
                                 ***********************
    Nge_Mir-1175                 ugagauucaacuccuccaacugc
    Lgi-Mir-1175_3p              ugagauucaacuccuccaacugc
    Lan-Mir-1175_3p              ugagauucaacuccuccaacugc
    Npo-Mir-1175_3p              ugagauucaacuccuccaacugc
    Cgi-Mir-1175_3p              ugagauucaacuccuccaacugc
    Cte-Mir-1175_3p              ugagauucaacuccuccaacugc
```
